# Highlights of the 16th St Gallen International Breast Cancer Conference, Vienna, Austria, 20–23 March 2019: personalised treatments for patients with early breast cancer

**DOI:** 10.3332/ecancer.2019.924

**Published:** 2019-04-24

**Authors:** Consuelo Morigi

**Affiliations:** Division of Senology, IRCCS European Institute of Oncology, 20141 Milan, Italy

**Keywords:** 16th St Gallen Consensus Conference 2019, early breast cancer, neoadjuvant, adjuvant, therapies, consensus

## Abstract

The 16th St Gallen International Breast Cancer Conference took place in Vienna for the third time, from 20–23 March 2019. More than 3000 people from all over the world were invited to take part in this important bi-annual critical review of the ‘state of the art’ in the primary care of breast cancer (BC), independent of political and industrial pressure, with the aim to integrate the most recent research data and most important developments in BC therapies since St Gallen International Breast Cancer Conference 2017, with the ultimate goal of drawing up a consensus for the current optimal treatment and prevention of BC.

This year, the St Gallen Breast Cancer Award was won by Monica Morrow (Memorial Sloan Kettering Cancer Center, USA) for her extraordinary contribution in research and practise development in the treatment of BC. She opened the session with the lecture ‘Will surgery be a part of BC treatment in the future?’ Improved systemic therapy has decreased BC mortality and increased pathologic complete response (pCR) rates after neoadjuvant chemotherapy (NACT). Improved imaging and increased screening uptake have led to detect smaller cancers. These factors have highlighted two possible scenarios to omit surgery: for patients with small low-grade ductal carcinoma in situ (DCIS) and for those who have received NACT and had a clinical and radiological complete response. However, considering that 7%–20% of `low-risk’ DCIS patients have co-existing invasive cancer at diagnosis, that surgery has become progressively less morbid and less toxic than some systemic therapies with a lower cost-effectiveness ratio, and that identification of pathologic complete response (pCR) without surgery requires more intensive imaging follow-up (more biopsies, higher cost and more anxiety for the patient), surgery still appears to be an essential treatment for BC.

The Umberto Veronesi Memorial Award went to Lesley Fallowfield (Brighton and Sussex Medical School, UK) for her important research and activity in the field of the development of patient outcome, of better communication skills and quality of life for women. In her lecture, she remarked on the importance of improving BC personalised treatments, especially through co-operation between scientists, always considering the whole woman and not just her breast disease. This award was given by Paolo Veronesi, after a moving introduction which culminated with the following words of Professor Umberto Veronesi:

‘It is not possible to take care of the people’s bodies without taking care of their mind. My duty, the duty of all doctors, is to listen and be part of the emotions of those we treat every day’.

## News and research priorities since St Gallen 2017

Regarding new developments in surgery, Walter Paul Weber (University Hospital Basel, Switzerland) spoke about the de-escalation of surgery of axilla according to the results of the IBCSG 23-01 and Z0011 trials [1, 2]. The current indications for axillary dissection (AD) are: clinically node-positive disease in upfront surgery, residual disease after NACT, locally advanced breast cancer (BC) [>2 pos sentinel lymph node (SN), gross extranodal disease, cT3-4, inflammatory] and SN macrometastasis in patient undergoing mastectomy (if post-mastectomy radiation is not indicated by the positive SN or does not include the regional node). The EORTC-AMAROS trial demonstrated that AD and axillary radiotherapy (ART) after a positive SN provide excellent and comparable axillary control for patients with T1-2 primary BC and no palpable lymphadenopathy, but with less morbidity by performing ART [3]. There are ongoing studies whose results will allow the extension of the Z0011criterial (SENOMAC, ERC/IPC). Currently, SN biopsy (SNB) after NACT in patients with initially positive nodes is accurate and reliable but requires patient selection and optimal surgical techniques [4–7]. The current ‘no ink on tumour’ for invasive BC was confirmed to be safe [8, 9].

Regarding radiotherapy (RT), Philip Poortmans (Institut Curie, France) underlined that the main research priorities are to adapt RT based on the individual patient’s risk factors: for low-risk women, use of a shorter treatment schedule (intra-operative RT) or only a few days of RT could be appropriate, on the contrary, for high-risk patients, fully locoregional RT should be considered. However, more research need to be done before speaking about truly personalised breast RT [10–13]. Something totally new appears to be the possibility of prophylactic irradiation of the contralateral breast for BRCA mutation carriers [14].

Martine Piccart-Gebhart (Institut Jules Bordet, Belgium) presented news about systemic therapies. In the adjuvant setting: ‘dose-dense’ chemotherapy reduces BC and all-cause mortality [15] and de-escalation chemotherapy with taxotere + cyclophosphamide (TC) × 6 could replace anthracycline-based regimens [16]. In luminal BC patients, de-escalation of chemotherapy (TAILORx - Trial Assigning IndividuaLized Options for Treatment (Rx)) appears to be possible. In pre-menopausal high-risk patients who have received chemotherapy, aromatase inhibitor (AI) and ovarian function suppression (OFS) are effective in reducing distant relapse at 8 years [17]. Extension of endocrine therapy (ET) beyond 5 years with an AI in postmenopausal women modestly affects recurrences; for some of these patients at high risk but with low compliance, intermittent administration of AI might be feasible with an improved quality of life [18]. In HER2pos high-risk BC patients, dual blockade improves invasive disease-free survival (IDFS) [19, 20]. De-escalation of chemotherapy by omitting anthracycline remains safe after 7 years in small volume disease (results from the APT trial), while de-escalating trastuzumab duration from 12 to 6 months appears still controversial [21]. There are many neoadjuvant trials to be considered, for instance, in luminal BC results derived from adding phosphoinositide 3-kinase inhibitor (PI3Ki) (LORELEI NEO-ORB) or cyclin-dependent kinase 4/6 inhibitor (CDK4/6i) (PALLET, NEOMONARCH) to AI are very interesting; in HER2pos BC, the high pCR using Trastuzumab emtansine (T-DM1) with or without ET (ADAPT) is noteworthy, and in another ADAPT trial, it is interesting that with pertuzumab + trastuzumab, the pathological complete response rate (pCR) is 36%, but adding docetaxel achieves a pCR of 90%. In triple negative breast cancer (TNBC), the role of platinum is still unclear. In the post-neoadjuvant setting, the KATHERINE trial should be mentioned: administration of T-DM1 (compared to trastuzumab) to patients with HER2pos BC showing residual disease at surgery following standard NACT and single or dual HER2-blockade revealed a striking 11% absolute improvement in IDFS. In TNBC and residual disease after NACT, the CREATE-X (Capecitabine for Residual Cancer as Adjuvant Therapy) trial demonstrated the benefit of post-neoadjuvant treatment with capecitabine.

Kathleen Pritchard (Sunnybrook Odette Cancer Center, Canada) stated that there are many research priorities in the treatment of BC women to better understand when and how to de-escalate or escalate treatments to guarantee the most accurate standard of care and the best quality of life for women.

## Biology of breast cancer: risk stratification

Richard Gelber (Dana-Farber Cancer Institute, USA) spoke about the fundamental role of staging and pathology in evaluating BC patients’ risk of recurrence and therapy selection [22–24]. Moreover, the morphological assessment of tumour-infiltrating lymphocytes (TILs) has an important prognostic role, in particular, in TNBC [25, 26] and HER2pos disease [27, 28]. Furthermore, the absence of pCR after NACT has showed the ability to stratify population for escalating adjuvant treatment among those patients with greater risk of recurrence and, on the contrary, de-escalating therapy amongst those with the lowest risk [29, 30]. The role of TILs in residual disease may further improve risk stratification [31].

Christos Sotiriou (Institut Jules Bordet, Belgium) focussed on the genomic risk stratification of early relapse. Gene expression profile (GEP) assays (e.g. Prosigna, Endopredict and Oncotype) have an important role in Estrogen Receptor (ER)pos/HER2neg BC as they inform the physician and patient on the risk of early recurrence and to assist them to choose the best treatment option in order to avoid recurrences but also useless treatments, in fact, this group of BC is a very heterogeneous disease and GEP might help to provide tailored treatment [32]. Data derived from studies such as TAILORx and MINDACT (Microarray in Node-Negative Disease May Avoid Chemotherapy) may be able to assess a prognostic relevance and help clinicians to evaluate the best treatment for every patient [33, 34]. Moreover, there are many ongoing studies assessing the predictive value of GEP for chemotherapy benefit in N1-2 BC (e.g. RxPONDER, WSG-ADAPT).

Regarding risk assessment of late recurrence from ERpos BC, Ivana Sestak (Wolfson Institute of Preventive Medicine, UK) reported that clinico-pathological parameters, especially nodal status and tumour size, are important for prediction of late recurrences. In some patients, extended ET can reduce recurrences but given the side effects of ET to define optimal patient selection for extended treatment is crucial. Many web-based risk calculators incorporating clinico-pathological and epidemiological parameters providing information on the risk of late recurrence have been developed, for example, the CTS5 calculator. Even if GEP have been developed to predict early recurrence, some of them have shown clinical validity for late recurrence too (e.g. BC Index, Prosigna and EndoPredict).

Fabrice André (Institut de Cancérologie Gustave Roussy, France) presented the argument ‘identifying research priorities in risk stratification of patients with BC’. Molecular tests could have the clinical utility to better decide which patients should receive chemotherapy or not, patients who can avoid ET with the rationale of not reducing quality of life unnecessarily [35], women at high risk even when optimally treated who are eligible for new (expensive) drugs and predict which patients will present outlier toxicity in order to substitute conventional treatments with new drugs. Regarding new tools for risk stratification, it should be taken into consideration that some genomic alteration could predict the outcome of BC patients—for instance, it has been reported in several studies that 8p11 amplification identifies a group of patients with poor outcome, in contrast, mutations on MAP3KA have been associated with better outcome [36]. Detection of circulating tumour DNA (ctDNA) after NACT and surgery could be associated with the worst prognosis [37], this tool could be important because we can detect micro metastases before to give local therapies. Testing pharmacodynamic of the drug *in vivo* [38] can also be relevant. Intratumour heterogeneity (genetic distance, lethal sub-clones in the primary tumour and genome instability) and genome evolution are going to give us information about the risk of relapse. Interestingly, there also appears to be a possible application for artificial intelligence and machine learning in this field of prediction [39].

## Biology of breast cancer: prediction of response

Aleix Prat (Hospital Clinic of Barcelona, Spain) reported in his presentation on the heterogeneity in treatment response in patients with HER2pos BC and the importance of combinations of clinical and biomarker data with well-designed trials in order to successfully escalate or de-escalate therapy. At the DNA level, the two most relevant somatic mutations within HER2pos disease are TP53 and PIK3CA; interestingly, these mutations tend to be mutually exclusive. PIK3CA mutant/HER2pos tumours had lower pCR in the neoadjuvant setting compared with wild-type tumours [40]. HER2-enriched intrinsic subtype, which represents 50%–60% of all HER2pos tumours, is an important biomarker tracking HER2-addition and chemotherapy sensitivity [41]. Tumour microenvironment is also important, in particular, TILs in the stroma is a consistent biomarker of better outcome [42]. TILs seem to be interesting also as an on-treatment biomarker, in fact, there is a correlation between increasing TILs during therapy and pCR [43].

Matthew J Ellis (Lester and Sue Smith Breast Center, USA) presented the difference in treatment response in luminal BC related to genomic complexity with extremely marked intra- and inter-tumoural heterogeneity. While many patients follow a benign clinical course, others manifest a lethal systemic disease with resistance to current ET which may due to: single-strand break repair defects which give adaptive evolution and drug resistance, specific DNA repair defects such as MutL defects (which, however, could be used to direct adjuvant CDK4/6i inhibitors) [44, 45] and specific gene mutation such as NF1 [46]. Interestingly, ESR1 fusion transcripts drive ET resistance and metastatic disease progression although CDK4/6i inhibitors are predicted to be effective [47]. Since the progression of ERpos BC is related to increased somatic mutation rates, immunotherapy approaches should be aggressively pursued.

Carsten Denkert (Charité University Hospital Berlin, Germany) stated that in the TNBC, there is heterogeneity of treatment response based on: high level of TILs which increase neoadjuvant response and improve prognosis [48], and PD-L1 (programmed death-ligand 1) which is a predictive biomarker expressed on the tumour cells, as well as on TILs in a subset of TNBC and has been shown to be predictive for response to a combination of immunotherapy and chemotherapy in the metastatic setting [49]. The current research challenges are the translations of biomarkers to early BC (e.g. GeparNuevo), integration of PD-L1 as a new marker for neoadjuvant and adjuvant clinical trials and selection of patients for additional therapeutic strategies.

Lisa A Carey (Lineberger Comprehensive Cancer Center, USA) underlined the research priorities in the prediction of response in early BC. HER2pos low-risk BC has excellent outcomes with paclitaxel + trastuzumab alone (APT trial) while a different situation appears in higher clinical risk HER2pos BC. Research priorities are the identification and the evaluation of biomarkers that could allow the best treatment for the patient [50–54]. In hormone receptor (HR)pos BC, it should be considered that pCR is meaningful but rare with treatments. The ALTERNATE trial (Estrogen Receptor positive breast cancer NeoAdjuvant. TrEatment) could provide the opportunity to prospectively validate a biomarker-driven strategy for treatment of ERpos postmenopausal women. MINDACT and TAILORx may clarify which patients can have benefits from chemotherapy. Moreover, multigene signatures (MGSs) can estimate the risk of late distant recurrence and this might help to choose the most appropriate treatments, such as, for instance, chemotherapy and/or extended ET for high-risk ERpos BC [55].

## Risk stratification and prevention in ductal carcinoma *in situ*

William F Symmans (MD Anderson Cancer Center, USA) presented the argument ‘risk stratification in patients with ductal carcinoma *in situ*’. It appears almost clear that women with higher grade ductal carcinoma *in situ* (DCIS), HRneg, larger size, have a significant risk of recurrence and even risk of invasive recurrence and require surgery associated with radiotherapy [56]. In ERpos DCIS, tamoxifen reduced the rate of ipsilateral and contralateral events [57].

Shelley Hwang (Duke University Hospital, USA) highlighted some current prospective randomised clinical trials regarding women with grade 1-2 DCIS with the challenge to reduce over-treatment (LORD, LORIS, COMET). Moreover, it is necessary to encourage effective communication between patients and their physicians about the pros and cons of treatment versus surveillance. Emerging molecular tools offer the potential to tailor treatment according to biology. Numerous efforts have evaluated the role of biomarkers in discriminating high from low-risk DCIS. Both genomic and proteomic approaches have been developed and appear promising, the results that will come from the PRECISION project seem to be very interesting in this area.

Andrea De Censi (Galliera Hospital, Italy) underlined a phase III trial, TAM01, which explores the role of low dose of tamoxifen ‘babytam’ (5 mg/day) in women who had been treated with surgery for breast intraepithelial neoplasia; this trial has demonstrated comparable risk reduction and significantly lower toxicity compared with data derived from trials of tamoxifen given at 20 mg/day (NSABP B-24, NSABP-P1). The expression of 23 genes involved in cell cycle progression (CCP score, Myriad Genetics) may provide prognostic and predictive markers to identify patients who can derive the greatest benefits from low dose of tamoxifen in terms of recurrences.

## Special lectures I

The first special lecture of the second day of the congress was held by Sharon H Giordano (MD Anderson Cancer Center, USA), who spoke about the necessity to extrapolate data from clinical trials to applying them in real life. It is very important to carefully select the study design, statistical techniques and also be aware of the limitations of trial data.

The second lecture was given by Ann H Partridge (Dana-Farber Cancer Institute, USA) and she spoke on the clinical benefit of treatments for women with BC, especially if these treatments convey current understanding of evidence and guidelines. It is important to consider individual patient preferences in order to determine the optimal treatment approach for every woman with BC: we should take care of the human being, not the disease.

## New pathways with potential impact in the treatment of early breast cancer

Andrew Tutt (The Institute of Cancer Research, UK) stated that homologous recombination (HR) deficiency deserves a lot of attention, in fact, it is significant both in family BC and in sporadic TNBC and it leaves a pathognomonic ‘scar’ on the genome and it may have important clinical benefits. Targeting this is now approved for two poly ADP ribose polymerase inhibitor (PARPi) for BRCA1-2 metastatic BC (EMBRACA, OLYMPIAD) while its role in adjuvant therapy will be clarified by the ongoing OlympiA trial. Moreover, it appears to be important to target HR in order to understand the mechanism of resistance and how treatment affects the evolution of resistance mechanisms. ATR inhibition can increase response and inhibit resistance to PARPi [58]. New strategies using combinations of PARPi with ATRi or Wee1i in advance TNBC may increase response and reduce resistance to treatment (VIOLETTE trial).

Nicholas C Turner (Royal Marsden Hospital, UK) focussed on the role of CDK4/6i and their important role in metastatic ER pos BC, both in association with AI (PALOMA2, MONALEESA2, MONARCH3) and with Fulvestrant (PALOMA3, MONARCH2). They have also demonstrated high activity (not really any evidence yet of efficacy improving outcomes) also in early BC, for instance when CDK4/6i are used preoperatively (POP, NeoPalAna, neoMONARCH, PALLET). Adjuvant studies (Penelope B, PALLAS, monarchE, NATALEE) have completed patient enrolment and we are awaiting the data and results from the follow up, in fact, early relapse in the first 2 years is not the main problem in ER pos/HER2 neg BC and longer follow up of all the studies will be critical. These studies between them are starting to help us to understand the most correct duration of treatment with CDK4/6i. Currently, emerging clinical biomarkers may predict the response to these drugs (e.g. mutations in RB1 and FAT1) but none of these are ready for clinical implementation.

Sherene Loi (Peter MacCallum Cancer Centre, Australia) spoke about target immunity in BC. TILs have shown strong prognostic value in early-stage TNBC treated with adjuvant chemotherapy [26]. High levels of TILs correlate with high level of PD-1, which is the predominant checkpoint present on both CD8+ and CD4+ T cells, whilst CTLA-4 is present on T regulatory cells. In contrast, the quantity of TILs in metastatic lesions is extremely low, indicating why advanced BC patients did not have high response rates to immune checkpoint PD-(L)1 inhibitors. Combinations of immunotherapy with chemotherapy have been investigated in the advanced setting (e.g. Impassion130). Checkpoint inhibitors are currently being investigated in the early stage setting in a number of phase II/III trials in TNBC with different anti-PD-1, PD-L1 and CTLA-4 agents. Trastuzumab resistance in HER2pos BC could be mediated by immune mechanism, adding pembrolizumab to trastuzumab showed a clinical benefit in HER2pos advance BC patients with PD-L1-pos, trastuzumab-resistant (PANACEA trial). In early-stage ERpos/HER2neg BC, TILs are associated with adverse biology, and in these patients, adding pembrolizumab seems to have an increase in pCR (I-SPY2). Further studies are necessary but target immune pathway in BC appears promising.

Fatima Cardoso (Champalimaud Cancer Centre, Portugal) presented on how the recent data from advanced BC (aBC) trials inform us on treatment opportunities for patients with early BC (eBC). It should be considered that surrogate endpoints do not consistently correlate with long term endpoints [59]. Moreover, when the benefit seen in aBC is substantial and includes overall survival (OS), usually the treatment is also useful in eBC, differently to when the benefit is moderate and/or only progression-free survival (PFS) is achieved, a substantial benefit in eBC is very rarely seen. What can we expect from the latest drugs approved for aBC and evaluated for eBC? Regarding CDK4/6i, a substantial PFS benefit was shown in the first line, a moderate PFS benefit in the second line, OS not statistically significant in the second line but probably because the trials were not sufficiently powered—it is likely that they have a future role in eBC. Regarding mammalian target of rapamycin inhibitor (mTORi)—moderate PFS benefit was demonstrated, OS not statistically significant (maybe also because the trials were not powered), with toxicity now better managed with the use of steroid mouth wash for stomatitis—they probably do not have a future role in eBC. This is likely to be the same for PI3Ki, currently only one of which has showed a moderate PFS benefit and all of these drugs showed too much toxicity. The role of Histone deacetylase inhibitor (HDACi) still remains uncertain: chidamide has a moderate PFS benefit but some toxicity and we are still waiting to hear about entinostat. Hopefully, PARPi, which currently shows small PFS benefit but important benefit in QoL, will have a future role in eBC. Regarding checkpoint inhibitors, we cannot conclude based on aBC trials the benefit for eBC because aBC and eBC are totally different in the immune setting.

## Treatment tailoring according to pathology and biology

Stefan Aeby (Luzerner Kantonsspital, Switzerland) presented the role of MGS. There isn’t a need for MGS for all patients (e.g. Oncotype Dx, Mammaprint, Endopredict and Prosigna), such as patients with an excellent prognosis based on conventional criteria and patients for whom management decisions will not be influenced by the results of MGS. For other patients, MGS scores may have a role in prognostication and prognosis models. TAILORx and MINDACT are examples of prediction prospective trials. Future studies may establish MGS to guide the choice of drug therapies with benefits for patients and also for health care systems avoiding unnecessary and costly treatments.

Hope S Rugo (University of California, San Francisco, USA) presented the treatment selection for patients with low and equivocal ER status. According to ASCO/CAP guidelines, if ER ≥ 1%, the term equivocal should not be used based on data, suggesting response to ET even in low ERpos (1%–9%). These groups of BC are relatively uncommon and heterogenous by gene expression profiling. Low HR expression is associated with higher KI67, higher grade and less progesterone receptor (PR) positivity, higher recurrence score and higher chemo-sensitivity. Regarding treatment recommendations, chemotherapy should be given following guidelines for TNBC [60]. Currently, the benefit of ET is unknown, but in general, it should be recommended despite the likely extremely small benefit, especially for lowest ERpos disease (<5%).

Giuseppe Viale (Istituto Europeo di Oncologia, Italy) focussed on the controversial treatment of patients with HER2 status. According to ASCO/CAP 2013 guideline recommendations, HER2 status is equivocal if both immunohistochemistry (IHC) (score 2+) and *in situ* hybridisation test for HER2 (4–6 copies of the gene and the ratio less than 2) are equivocal. ASCO/CAP 2018 recommended that these cases must be considered HER2neg due to the lack of evidence for any benefit from HER2-targeted therapy in these groups of patients as the results of the B-47 trial suggest. It is important to consider the intra- and inter-tumoural heterogeneity and the fact that there is no correlation between IHC and gene expression profiling. Knowing this is important, for example, in order to de-escalate therapy, in fact, in the neoadjuvant setting without chemotherapy we may have: in HER2pos/HRneg with dual blockade, 20%–30% of pCR, in HER2pos/HRpos with lapatinib + trastuzumab + letrozole, 21% of pCR (TBCRC006 trial) and 32% of pCR with lapatinib + trastuzumab + ET (PAMELA). In the adjuvant setting, however, the larger benefit from trastuzumab in HRneg compared to HRpos disease has not been observed and all the intrinsic subtypes did benefit similarly from trastuzumab. Also, with adjuvant T-DM1 for residual disease after neoadjuvant therapy, there is evidence of interaction between HR status although there is likely better response of HER2pos/HRpos disease to extended therapy with neratinib (ExteNET - Extended Adjuvant Treatment of Breast Cancer with Neratinib). In the advanced setting, expression of ER > 30% of tumour cells is associated with reduced probability of response to chemotherapy and trastuzumab, maintenance ET is associated with a significant reduction in the risk of progression and dual blockade of HER2 with an AI is effective for treatment of HER2pos/ERpos disease in the first line (PERTAIN trial). Possible new treatment options include: triple blockade of ER, HER2 and CDK4/6 both in the neoadjuvant setting (NAPHER 2) and in the advanced setting (PATRICIA) and anti-immune checkpoint agent in the neoadjuvant setting (APTneo, neoHIP trial).

Judy E Garber (Dana-Farber Cancer Institute, USA) spoke about treatment selection for patients with BRCA mutations that are more predisposed to BC by impairing HR and causing genomic instability. HR also repairs DNA lesions caused by platinum agents and PARPi, thus these groups of patients have increased sensitivity to them. According to the results of the POSH trial, BRCA status should not be viewed as an independent poor prognostic factor. Some studies demonstrated major sensitivity to carboplatin [61] but in others, it is less evident (GeparSixto). Additional prospective studies stratified by BRCA1 and BRCA2 mutation status are needed to better understand the effect of carboplatin in polychemotherapy regimens. It should be considered in fact, that the BRCA mutation carriers make the tumour more sensitive to killing by chemotherapy that induces DNA damage [62]. PARPi were approved for treatment of metastatic BC with BRCA mutations (OlympiAD, Abrazo, EMBRACA). Lurbinectedin is another drug that targets DNA repair, it showed an interesting activity in patients with BRCA mutation [63]. There are ongoing studies evaluating combination therapies such as PARPi with immunotherapy (MEDIOLA). The phase III study BRCA-P will determine if denosumab may have a role in the prevention of BC in BRCA1 mutation carriers.

## Surgery of early breast cancer

Paolo Veronesi (Istituto Europeo di Oncologia, Italy) started the surgical session with a presentation based on standards and controversies in SN, making a quick historical explanation from the initial surgical approach to the axilla with the AD for all patients with BC, passing on the SNB, to the current management. Nowadays, the axilla treatment does not require AD for all BC patients, for example women who are candidates for BC conservative surgery macrometastases in 1-2 SN (Z0011 trial), patients undergoing mastectomy with micrometastatic SN (IBCSG 2301) and patients who underwent NACT and shifted from a status of cN1 to cN0. Moreover, data derived from the SOUND trial will indicate whether it may avoid SNB in patients with clinical and radiological negative axilla. Data derived from ongoing studies (SENOMAC) may further reduce axillary surgery in the future, but nowadays even if medical treatment recommendations depend mostly on primary tumour biology rather than on axillary status, information about axillary status given by SNB still remains important.

Bahadir Gulluoglu (Marmara University School of Medicine, Turkey) focussed his presentation on the impact of older age on local treatment decisions. Systematic analyses showed that both ET alone and surgical approach plus adjuvant ET provided similar OS rates, but without surgery, there is a higher local recurrence (LR) rate [64]. The surgical option remains the standard in physiologically fit elderly patients with all subtypes of BC, but in fragile HRpos BC patients with limited life expectancy, it may be possible to consider primary ET, with AI being preferable over tamoxifen [65]. Controversy also remains regarding the utility of whole breast irradiation (WBI). WBI reduces LR rate but does not improve OS, and therefore it can be omitted in unfit patients or in fit patients with clinical low-risk BC [66, 67]. SNB can possibly be omitted in unfit patients and in fit patients with clinical low risk or in those in which SN information will not change surgical management [68, 69]. In summary, it is not the age which governs the outcome of BC but the biology of disease (which is often more favourable in elderly patients), however, in this group of patients, it is necessary to assess multiple considerations to decide the best treatment.

Emiel JT Rutgers (The Netherlands Cancer Institute, The Netherlands) presented the controversial argument of prophylactic mastectomy in women without BRCA mutation. In this group of patients, a possible contralateral prophylactic mastectomy (CPM) should be a shared decision between women and their physicians. CPM has not shown any survival benefit, the annual risk of contralateral BC (second primary) is only 0.3%–0.5%, breast conservative surgery is as oncologically safe as mastectomy and CPM may have side effects that can lead to further surgery or adverse effects on body image and quality of life [70–74]. Moreover, in women < 40 years, with an extensive family history and a germline mutation in CHEK2 (11000delC), it is important to intensify screening with an annual mammography starting from 35 years [75]. In some case, CPM could be justified, especially in patients < 30 years or < 40 years with very dense breasts or extensive family history, CHEK2 mutation and high levels of anxiety and fear.

Florian Fitzal (Medical University Vienna, Austria) stated that breast conservative surgery is safe in new borders after NACT but care should be taken with large or multifocal disease and luminal subtype with more possibility of residual scatter cells over original tumour volume [76]. There is no difference in terms of LR comparing different mm of R0 resection after NACT, the gold standard is ‘no ink on tumour’ [77]. The resection of the whole residual microcalcification is still necessary after NACT [78]. Currently, omitting surgery after NACT is still not possible, but there are many ongoing trials that will perhaps lead to a change in this approach. The optimal time after last NACT and surgery is 21 days: surgery within 21 days was associated with an improved OS [79]. There are many ongoing trials that will clarify the standard of care of axillary surgery (Alliance A11202, SAKK 23/16, IBCSG 57-18 and ABCSG-53).

## Radiotherapy for early breast cancer

Timothy J Whelan (McMaster University, Canada) remarked that breast irradiation in some patients may be omitted. Nowadays, there are many trials evaluating the omission of RT in patients at low biological risk based on biomarker assessment (e.g. LUMINA, PAM50, PRIMETIME). These trials will help to quantify the risk of LR and select patients for whom RT may be omitted. There are also trials evaluating the elimination of regional nodal radiotherapy in patients with limited node-positive disease and low biological risk (e.g. MA.39).

Harry Bartelink (The Netherlands Cancer Institute, The Netherlands) presented the clinical benefit of regional node irradiation (RNI) for BC. Meta-analyses showed that post-mastectomy RT is effective and reduced both LR and BC mortality in women with SNpos [80]. Three recently published prospective trials (DBCG, EORT and MA.20) showed a reduction in BC mortality and recurrences with RNI. RNI after AD is recommended in high-risk patients with N1-3 positive nodes and if positive nodes are more than 3. In the AMAROS trial, both AD and ART provide a comparable LR in SNpos patients but the lymphoedema was doubled after AD compared to ART. The IBCSG 23-01 [1] showed that there is no need for extra treatment of the axilla after micro-metastases in SN. New data are expected from ongoing trials about SNpos after NACT (Alliance A011202, NSABP B-51/RTOG 1304).

Boon H Chua (Prince of Wales Hospital, Australia) focussed attention on the individualization of RT after surgery. Whole breast radiotherapy (WBRT) after breast-conserving surgery improves local control and BC survival. However, hypofractionated WBI (HF-WBI) also showed equivalent tumour control (START-P, START-A, START-B, OCOG); the use of HF-WBI in under-represented patient subgroups (e.g. very young patients) remains controversial. There are randomised clinical studies of HF-WBI involving a 1-week period (FAST, FAST-forward) that may well improve the balance of local control and side effects. Tumour bed boost (TBB) is especially used in higher-risk patients and improved local control but also increased breast fibrosis (EORTC, Budapest, Lyon). The IMPORT HIGH trial compared sequential TBB and simultaneous integrated TBB given during WBRT and it showed similar toxicity but efficacy data are still pending. There are many trials involving partial breast irradiation (PBI), some of these, such as IMPORT LOW and RAPID, showed that PBI resulted in low absolute LR rates similar to WBI but higher rates of late toxicity and adverse cosmetic outcome. Other PBI trials, however, have highlighted higher LR rate compared to WBI [81]. Current data support PBI use for selected low-risk patients but longer-term safety data are essential for definitive evaluation. Regarding clinical trials demonstrating the efficacy of PMRT: results derived from trials suggest that chest wall is an important target volume, while current evidence does not enable the distinction of relative contributions of nodal target sub-volumes to treatment efficacy, routine RT of internal mammary chain is controversial but ASCO-guidelines recommend that internal mammary chain (IMC) should be included in patients with SNpos BC receiving PMRT.

The use of hypofractionated-PMRT currently remains unclear. Patients with breast reconstruction are at increased risk of late fibrosis and adverse cosmetic outcome after RT but there are no current guidelines for target volume definition after reconstruction.

John H Maduro (University Medical Centre Groningen, The Netherlands) spoke about the potential role of proton irradiation compared to conventional photon irradiation. Protons are charged particles able to deliver the dose to a specified depth where they stop, with a reduction of cardiac and pulmonary toxicity [82]. Patients who could benefit from this technique for excellent local control, good cosmetic results and low dose given to mediastinal organs are: young patients, bilateral BC, patients with cardiac risk factor or special anatomy (pectus excavatum) and patients who need IMC irradiation [83]. Nowadays, however, even if there has been a clear increase in proton facilities in recent years, the availability remains scarce and the costs high.

## Special lecture II

In his lecture, Eric P Winer (Dana-Farber Cancer Institute, USA) presented principles and practical considerations about systematic treatments. The goals of adjuvant therapy are: eradicate micrometastatic disease, improve OS, and delay recurrence resulting in a better overall quality of life. The goals of neoadjuvant therapy are: eradicate micrometastatic disease, improve OS, decrease extent of surgery, provide prognostic information, select candidates for additional treatment, test de-escalation trials and strategies and conduct tissue-intensive trials. Adjuvant chemotherapy with upfront surgery should be preferred, dependent on the anatomic extent of disease and when it is impossible to follow disease during NACT. Neoadjuvant treatment should be preferred in stage II/III TNBC or HER2pos disease, in stage II/III ERpos, if it is clear that chemotherapy will be administrated and if optimal surgery treatment will be facilitated by NACT. Even neoadjuvant ET should sometimes be considered, for instance: women who are not considered to have chemotherapy sensitive BC, to optimise breast conservation in stage II/III disease, to assess prognosis through assessment of KI67 or other markers and determine the need for chemotherapy (but this is still being investigated). pCR is important but is a strong predictor for individual patients and a poor predictor of the long term success of regimen in terms of OS and DFS [84]. It is time for a new approach for clinical trials in the neoadjuvant setting which can lead to individualization therapy and improved outcomes (COMPASS trial).

## Primary and adjuvant systemic therapy of early breast cancer: estimating the magnitude of clinical benefit

Angelo Di Leo (Azienda USL Toscana Centro, Italy) discussed chemotherapy in luminal BC. This group of BCs has generally favourable prognosis, but sometimes some of them have a poor prognosis. In the future, we could benefit from the clinical use of circulating tumour cells (CTC) to assess prognosis [85, 86]. Circulating tumour DNA (ctDNA) methylation in serum and plasma has been shown to be effective for diagnostic or prognostic biomarkers detection in different tumour types and in the future will be useful in clinic. DNA methylation alterations across the different tumour types, in fact, may occur more frequently than genomic alterations on ctDNA and apparently could be a more sensitive technology with respect to gene mutations on ctDNA, moreover, some DNA methylation seems to be less dependent on BC subtypes compared to gene mutations [87]. There is emerging evidence that metabolomic profiling may have the ability to further stratify risk within existing genomic assay determined risk categories [88, 89]. Integrating standard pathology and genomics of primary tumour with tools detecting ‘signals’ of active micro-metastatic disease (blood, serum and plasma) is a challenge for new prospective designed studies in the attempt to produce better prognostic stratification of eBC.

Prudence Francis (Peter McCallum Cancer Centre, Australia) spoke about adjuvant ET in premenopausal BC patients. According to the data, the use of adjuvant tamoxifen (T) for 5 years in these women determines substantial reductions in recurrence with an important improvement of OS. Updated results from the suppression of ovarian function trial (SOFT) and tamoxifen and EXemestane trial (TEXT) have helped to identify the differential value of different adjuvant ET strategies in high-risk and low-risk premenopausal women but longer follow-up remains fundamental. With a median follow-up of 8 years in SOFT, there is a significant improvement in DFS for patients assigned to receive T + OFS for 5 years as compared with T alone for 5 years. Further improvement in DFS was observed for those assigned 5 years’ of exemestane + OFS (AI + OFS). While these results applied to the overall randomised SOFT premenopausal population, larger absolute improvements in DFS were observed with ET escalation in the premenopausal cohort who had received prior chemotherapy due to the perceived risk of recurrence, while smaller absolute improvements in DFS were observed in women who did not receive chemotherapy. Despite some improvements in DFS in the lower risk no-chemotherapy cohort, the SOFT 8-year results for that cohort continue to demonstrate a low risk of recurrence or death with the use of adjuvant tamoxifen alone. Small OS advantages from the addition of OFS to T have now emerged in SOFT, which appear more clinically meaningful in the cohort who received prior chemotherapy. In the joint analysis of the SOFT and TEXT trials, there is currently no OS improvement at 8 years for those assigned AI + OFS versus those assigned T + OFS despite a significant reduction in distance recurrences with AI + OFS. In women < 35 years if AI + OFS are not tolerated, may be consider T + OFS before T alone in fact, in the SOFT trial after median 8 years, among those patients assigned T more than 1/3 had an invasive BC and more than 1/4 had a distant recurrence. There is evidence to support 5 years of OFS + AI/T but, according to the ATLAS (Adjuvant Tamoxifen Longer Against Shorter) trial, continuing tamoxifen up to 10 years produces an addition to a statistically significant improvement of OS and reduction of recurrence and BC-specific mortality. In choosing the duration of ET, the following should be considered: baseline tumour risk, age, tolerance to ET, and fertility.

Harold J Burstein (Dana-Farber Cancer Institute, USA) focussed attention on adjuvant ET in postmenopausal women. The current treatment options include T, AI or a sequence of these. Individual trials and meta-analysis suggest that AI treatment, such as either initial or sequential therapy, reduces recurrence risk compared to 5 years of T alone and it appears to be better, especially for women with higher risk tumours and lobular carcinomas (BIG 1-98, ABCSG 8). Extending ET (NSABP B-42, MA.17, DATA) reduces the risk of recurrence. It is important to consider that ET also have side effects which can be reduced by switching a type of ET with another one or, for example, regarding arthralgia, using omega-3 fatty or acupuncture [90, 91]. The aim for better therapeutic management is to identify patients with intermediate-high risk of recurrences that could potentially benefit from the extension of ET, genomic signature has been shown to be prognostic in term of the risk of recurrences [92]. Currently, we should consider the extension of ET in women with high stage BC, especially patients who have tolerated the treatment and are willing to continue, and patients who started with T [93].

Robert Coleman (Weston Park Hospital, UK) focussed on the clinical benefit of bisphosphonates, which should be part of routine treatment in the adjuvant setting in postmenopausal women with early BC at significant risk for disease recurrence. In fact, as highlighted, for example, in the EBCTCG Meta-analysis, bisphosphonates reduce the development of bone metastases and improve survival and should be given to postmenopausal patients with intermediate/high risk [94]. Benefits appeared similar across different bisphosphonates tested suggesting a class effect. These effects could not be reproduced in the large adjuvant trial of denosumab (DCARE). However, this drug has efficacy in prevention of fractures associated with the use of AI and so, for patients at low risk for recurrence, it may be preferred over a bisphosphonate for the preservation of bone health. Biomarkers as MAF deserve further investigation and may aid selection of patients for adjuvant target treatments, for example, in the AZURE trial, it has been seen that amplification of MAF predicts an adverse effect of zolendronic acid in terms of DFS and OS in pre/perimenopausal women, on the contrary, patients with MAF negative tumours benefited from this treatment irrespective of menopausal status [95].

Nadia Harbeck (Comprehensive Cancer Center of the Ludwig-Maximilians-University, Germany) underlined emerging strategies in the neoadjuvant treatment of HER2pos BC. Future research need to focus on avoiding over-treatment of patients with pCR which is a prognostic factor for both DFS and OS in HER2pos BC (TECHNO). De-escalation therapeutic strategy should involve reducing the chemotherapy component while optimising anti-HER2 therapy; on the contrary, escalation should take into consideration immunotherapy and novel HER2-targeting drugs.

Ian E Krop (Dana-Farber Cancer Institute, USA) presented the optimisation treatment for HER2pos BC. With the use of HER2 directed therapies, outcomes for patients with early-stage HER2pos BC are now favourable for all but the highest risk women (APHINITY, APT). These favourable outcomes increase the importance of risk stratification to minimise over and under treatment, and de-escalation strategies to potentially further reduce the toxicities of therapy. Tumour size, lymph node status and HR status have well-established prognostic value in HER2pos BC, and these can be utilised to risk stratify patients. However, one of the strongest prognostic markers in HER2pos BC is pcR after NACT (I-SPY). Thus, treating patients in the neoadjuvant setting provides prognostic data and can identify patients who may benefit from a change in therapy, just think of T-DM1 for patients without pCR (KATHERINE). Currently, there are no data comparing T-DM1 to trastuzumab + pertuzumab in the adjuvant setting. However, given the large benefit seen in KATHERINE compared to the relatively small benefit of the addition of pertuzumab in APHINITY, it would seem unlikely that adjuvant T-DM1 would be inferior to the other treatment. The ExteNET study demonstrated a benefit to a year of neratinib in ERpos/HER2pos BC completing a year of (neo)adjuvant HER2 directed therapy. Is there a role for neratinib in patients who received T-DM1 after neoadjuvant therapy? As there are no data on adjuvant neratinib after T-DM1 or pertuzumab, it is difficult to recommend neratinib in patients after T-DM1. However, in the absence of such data, neratinib can be considered in rare cases with extremely high-risk ERpos BC patients after a year of T-DM1. In patients with low clinical risk HER2pos BC, 12 weekly doses of paclitaxel with a year of trastuzumab demonstrate very good outcomes: in this subgroup of women neoadjuvant treatment can be avoided (APT).

Javier Cortes (Vall d’Hebron Institute of Oncology, Spain) spoke about neoadjuvant and adjuvant chemotherapy in TNBC. TNBC is a very heterogeneous group of tumours in terms of genetic profile and in histology. Clinical trials (CALGB 40603, GeparSixto) evaluated the addition of platinum compounds in the neoadjuvant setting and demonstrated an increase of pCR but not a statistical improvement of DFS or OS. For some patients at high risk, platinum may be taken into consideration. The role of adding nab-paclitaxel is still unclear with different results from the data (GeparSepto, ETNA). Instead, using both nab-paclitaxel and platinum without anthracyclines in the neoadjuvant setting produces 50% pCR and may be considered in clinic, for example, in patients with allergic reactions (ADAPT trial). The role of PARPi is uncertain, in fact, in I-SPY2 (which resulted in higher rates of pCR compared to standard therapy), bot veliparib and carboplatin have been added to standard therapy. Interesting data derived from the use of talazoparib alone in advanced BC with germline BRCA1/2 mutation, with a significant benefit over standard chemotherapy [96]. Immunotherapy shows promising activity with upcoming trials such as KEYNOTE-522 and GeparDouze. In the adjuvant setting, chemotherapy should be started as soon as possible after surgery; however, delayed treatment has a big negative impact on TNBC [97]. In the post-neoadjuvant setting, capecitabine for high-risk patients with residual disease after NACT should be considered (CREATE-X), maybe also for patients pre-treated with platinum compounds. The results which will come from ECOG-ACRIN EA1131 will be interesting, a phase III trial that investigates the role of adjuvant platinum in patients with no pCR after NACT. Biology-driven clinical trials in residual tumours will be key to optimise new strategies in TNBC.

Antonio C Wolff (Johns Hopkins University, USA) concluded with a presentation that remarked on the advances in adjuvant therapies and greater access to care which have resulted in a steady improvement in survival outcomes for BC patients across the world. However, it is necessary to consider the short-term toxicities, as well as the long-term ones, which determine a worsening of quality of life [98]. In these terms, the correct management of therapy and also integrative therapies which can help to reduce the impact of the side effects assume great importance [99]. Non-medical factors, like underemployment, socioeconomic status and ‘financial toxicity’ can also negatively impact on health outcome and quality of life [100]. Nowadays, it is important to reduce barriers to care as much as possible. Therapies should be effective, offer value, result in meaningful outcomes and be available.

## Conclusions

The 16th St Gallen International Breast Cancer Conference did not disappoint the delegates’ high expectations. The presentations of major international experts in the different fields of therapeutic application gave the impression of more curable BC with increasing optimism for the future through the integration of strategies of care.
